# Mitochondrial microsatellite instability in patients with metastatic colorectal cancer

**DOI:** 10.1007/s00428-015-1733-8

**Published:** 2015-02-20

**Authors:** S. Venderbosch, S. van Vliet, M. H. C. Craenmehr, F. Simmer, A. F. J. de Haan, C. J. A. Punt, M. Koopman, I. D. Nagtegaal

**Affiliations:** 1 Department of Pathology, Radboud University Medical Center, PO Box 9101, 6500 HB Nijmegen, The Netherlands; 2 Department for Health Evidence, Section Biostatistics, Radboud University Medical Center, Nijmegen, The Netherlands; 3 Department of Medical Oncology, Academic Medical Centre, University of Amsterdam, Amsterdam, The Netherlands; 4 Department of Medical Oncology, University Medical Centre Utrecht, Utrecht, The Netherlands

**Keywords:** Metastatic colorectal cancer, mtMSI

## Abstract

Mitochondrial microsatellite instability (mtMSI), a change in length in mtDNA microsatellite sequences between normal and tumor tissue, has been described as a frequent occurrence in colorectal cancer (CRC). We evaluated the prevalence and prognostic value of mtMSI and its relation to nuclear microsatellite instability (MSI) in patients with metastatic CRC (mCRC). At six loci (D310, D514, D16184, ND1, ND5, and COX1), the mitochondrial DNA sequence was analyzed in normal and tumor tissue, and the mtMSI status was determined. We evaluated the prevalence and outcome in terms of overall survival (OS) in 83 CRC patients with a MSI tumor (including 39 patients with Lynch syndrome) and in 99 mCRC patients with a microsatellite stable (MSS) tumor. A meta-analysis was performed to compare our findings with existing data. mtMSI at the D-loop region was found in 54.4 % (99 out of 182) of all patients. Prevalence of mtMSI was most pronounced at the D310 locus (50.5 %). Prevalence of mtMSI at the D-loop region was not different among patients with MSI compared to MSS tumors. There was no effect of mtMSI on prognosis in patients with MSI or MSS tumors. Prevalence of mtMSI was high in mCRC patients with both MSI and MSS tumors, but there was no correlation with prognosis. mtMSI was particularly present at the D310 locus.

**Electronic supplementary material:**

The online version of this article (doi:10.1007/s00428-015-1733-8) contains supplementary material, which is available to authorized users.

## Introduction

Colorectal cancer (CRC) carcinogenesis is a multistep process involving the accumulation of genetic changes. CRC research is largely focused at activation of oncogenes, inactivation of tumor suppressor genes, and defects of mismatch repair genes in nuclear DNA. However, there are still many aspects that cannot be explained by these genetic changes.

More than half a century ago, Otto Warburg was the first to describe that mitochondria may play a role in carcinogenesis [47]. A mitochondrion is a membrane-enclosed intracellular organelle present in many copies per cell, which contains its own genetic system for replication, transcription, and translation [44]. Mitochondria generate most of the cell’s supply of adenosine triphosphate (ATP), the major energy source of the cell, and are therefore involved in cell signaling, cellular differentiation, apoptosis, and control of the cell cycle and cell growth.

The mitochondrial DNA is a 16,569-base pairs, double-stranded, circular DNA composed of genes and a noncoding region, the displacement loop (D-loop), which contains essential transcription and replication elements [30, 45]. The genes encode for both a small (12S) and a large (16S) ribosomal RNA gene, 22 transfer RNAs and 13 proteins.

The genetics of mitochondria differ from genetics of the nuclear genome in 3 major characteristics: maternal inheritance, heteroplasmy, and mitotic segregation [8]. Initially, it was assumed that mitochondrial DNA is homoplasmic in normal cells, that is, all of the mitochondrial DNA copies are identical not only in an individual cell but also among cells. However, new sequencing techniques revealed that heteroplasmy is not restricted to cancer cells but can also be present in normal tissue [15].

It has been reported that mitochondrial DNA is more susceptible to mutations than nuclear DNA, due to the lack of histones and chromatin structure, paucity of introns, inefficient mitochondrial DNA repair mechanisms and a higher exposure to reactive oxygen species (ROS) produced by ATP synthesis [33, 41]. Several hotspots of mitochondrial DNA mutations have been described in tumor tissue. A major target was found at the D310 sequence (C_n_TC_6_) in the non-coding D-loop [37]. In CRC, somatic mutations were also found at the D514 and D16184 sequence in the non-coding D-loop, rRNA genes, NADH dehydrogenase subunits (ND1, ND4L, and ND5), cytochrome *b*, and cytochrome oxidase subunits (COX1, COX2, and COX3). The majority of mutations were nucleotide substitutions or single base pair insertions. However, only few studies have sequenced the entire mitochondrial genome [15, 23]; most studies focused only on a few regions or exclusively at the D-loop region in CRC [1, 2, 9, 16, 17, 19, 40, 46]. Data about mitochondrial DNA mutations are therefore scarce and inconsistent.

Mitochondrial microsatellite instability (mtMSI) has been defined as change in length in short base repetitive sequences of mitochondrial DNA (mtDNA) between normal and tumor tissue. mtMSI has been described as a frequent occurrence in human cancers [4, 5] and also in CRC [12, 14, 35]. Several studies have evaluated the relation of nuclear MSI with mtMSI, but results are inconclusive. The association between mtMSI and other clinical factors was also analyzed. In stage III CRC cancers, the presence of mtMSI at the D-loop is associated with poor prognosis and resistance to fluorouracil-based adjuvant chemotherapy [27].

To date, no data are available regarding the prevalence or prognostic value of mtMSI in metastatic CRC (mCRC) patients. We hypothesize that the presence of mtMSI might confer a worse prognosis in mCRC, as seen in stage III CRC. We therefore assessed the role of mtMSI in respect to prevalence and outcome in patients with mCRC with either MSI or microsatellite stable (MSS) tumors.

## Materials and methods

### Clinical samples

Data were derived from mCRC patients included in two large phase III studies: CAIRO (Clinicaltrials.gov NCT00312000) and CAIRO2 (Clinicaltrials.gov NCT00208546), of which the results have been published previously [21, 42]. Collection of formalin-fixed paraffin-embedded (FFPE) material of the primary tumor was part of the initial protocol in both studies. To check that the normal colon tissue of CRC patients has a constant sequence pattern, we selected 10 patients from our hospital database for which we could obtain normal tissue from proximal and distal locations relative to the tumor.

In order to analyze the prevalence and relation between nuclear MSI and mtMSI, we selected 44 mCRC patients with MSI tumors from the CAIRO studies (CAIRO [22], *n* = 19, and CAIRO2 [42], *n* = 25) and 39 CRC patients with a known Lynch syndrome from our own database. Furthermore, we selected 56 mCRC patients with MSS tumors treated in the CAIRO study who were matched for known prognostic factors (the ‘test group’); all patients that were treated with first-line capecitabine monotherapy for at least 3 cycles had a resection of the primary tumor, WHO performance score 0, normal serum lactate dehydrogenase (LDH) concentrations, localization of the primary tumor in colon or rectosigmoid and had not received prior adjuvant chemotherapy. In order to validate the results found in the test group, we randomly selected 43 patients with MSS tumors treated in the CAIRO study as a validation group.

### Mitochondrial microsatellite instability (mtMSI) analysis

Genomic DNA was extracted from four to eight manually microdissected 30-μm sections of FFPE tissue. Areas containing >50 % tumor cells were selected by microscopic evaluation on a reference slide stained with H&E. Genomic DNA from microdissected tissues was isolated using the QIAamp® DNA micro kit (Qiagen, Valencia, CA) following the manufacturer’s instructions. DNA concentration was determined at 260 nm using the Nanodrop ND-1000 spectrophotometer (Nanodrop Technologies, Inc., Wilmington, DE, USA). The sequence of mutational hotspot regions was determined by PCR and Sanger sequencing using six primer sets: D-loop 310 (C_8_TC_6_), D-loop 514 (CA_5_), D-loop 16184 (C_12_), ND1 (C_6_), ND5 (C_6_A_8_), and COX1 (A_7_) (Supplemental Table 1) [10, 25, 28, 43]. The fragments were analyzed for length variations in tumor tissue compared to normal tissue (instability). We read the electropherograms manually to identify variable sequence length present at low level and to determine if there was coexistence of different subpopulations of the mononucleotide tracts in tumor tissue and normal tissue (heteroplasmy). Heteroplasmy makes analysis with GeneScan, which is used for nuclear MSI, difficult. Some samples revealed a different proportion of mtDNA subpopulation between normal and tumor tissue. Such a difference can occur due to changes in the proportion of mtDNA subpopulations during mitochondrial segregation or due to mutations, and we also classified these as instable. The analyses were performed in duplicate on normal and tumor DNA of the selected patients. The results were independently scored by two observers. In case of inconsistent results, the entire mtMSI experiment was repeated, and the final conclusion was based on two similar results. Due to technical failure, only 152 instead of 182 patients were analyzed for ND5.

### Statistical analysis

Patients were analyzed for the prevalence and prognostic value in four different groups: patients with combined MSI and mtMSI tumors (MSI/mtMSI), patients with combined MSI and mtMSS tumors (MSI/mtMSS), patients with combined MMS and mtMSI tumors (MSS/mtMSI), and patients with combined MSS and mtMSS tumors (MSS/mtMSS). The prevalence of mtMSI was compared for patients with MSI and MSS tumors. The outcome was analyzed within the group of patients with MSI tumors (excluding the Lynch syndrome patients) for mtMSI compared to mtMSS tumors and within the group of patients with MSS tumors for mtMSI compared to mtMSS tumors.

For the mtMSI analysis of the D-loop region (D310 locus, D514 locus, and D16184 locus) and per loci, patients were divided into two groups: stable versus instable tumors. The comparison of the prevalence of mtMSI between groups (MSI versus MSS) was performed by chi-square or Fisher’s exact tests, where appropriate. OS was defined as the time from randomization until death from any cause. OS curves were estimated using the Kaplan–Meier method and compared using a Cox proportional hazard model. All tests were two-sided, and *p* < 0.05 was considered as statistically significant. All analyses were conducted using the SAS system version 9.2.

### Literature search strategy, inclusion criteria, and data extraction

We reviewed the literature on the prevalence of mtMSI in stage I–IV CRC patients. The primary outcome of interest was prevalence of mtMSI. A search was conducted of Medline, PubMed, and the Cochrane Library from January 1998 (year of the first publication of mtMSI in CRC) to January 2014 with an English-language restriction using the following search terms: mitochondrial microsatellite instability, mitochondrial mutations, and mitochondrial alterations, in combination with colon cancer and colorectal cancer. Original publications were selected if the abstract contained data for patients with mtMSI. In case of duplicate publications, the most recent and/or most complete study was included. We excluded publications of patients with colon cancer and CRC in which only mutations in the mitochondrial genome were described.

## Results

### Prevalence of mtMSI at the D-loop

**Fig. 1:**
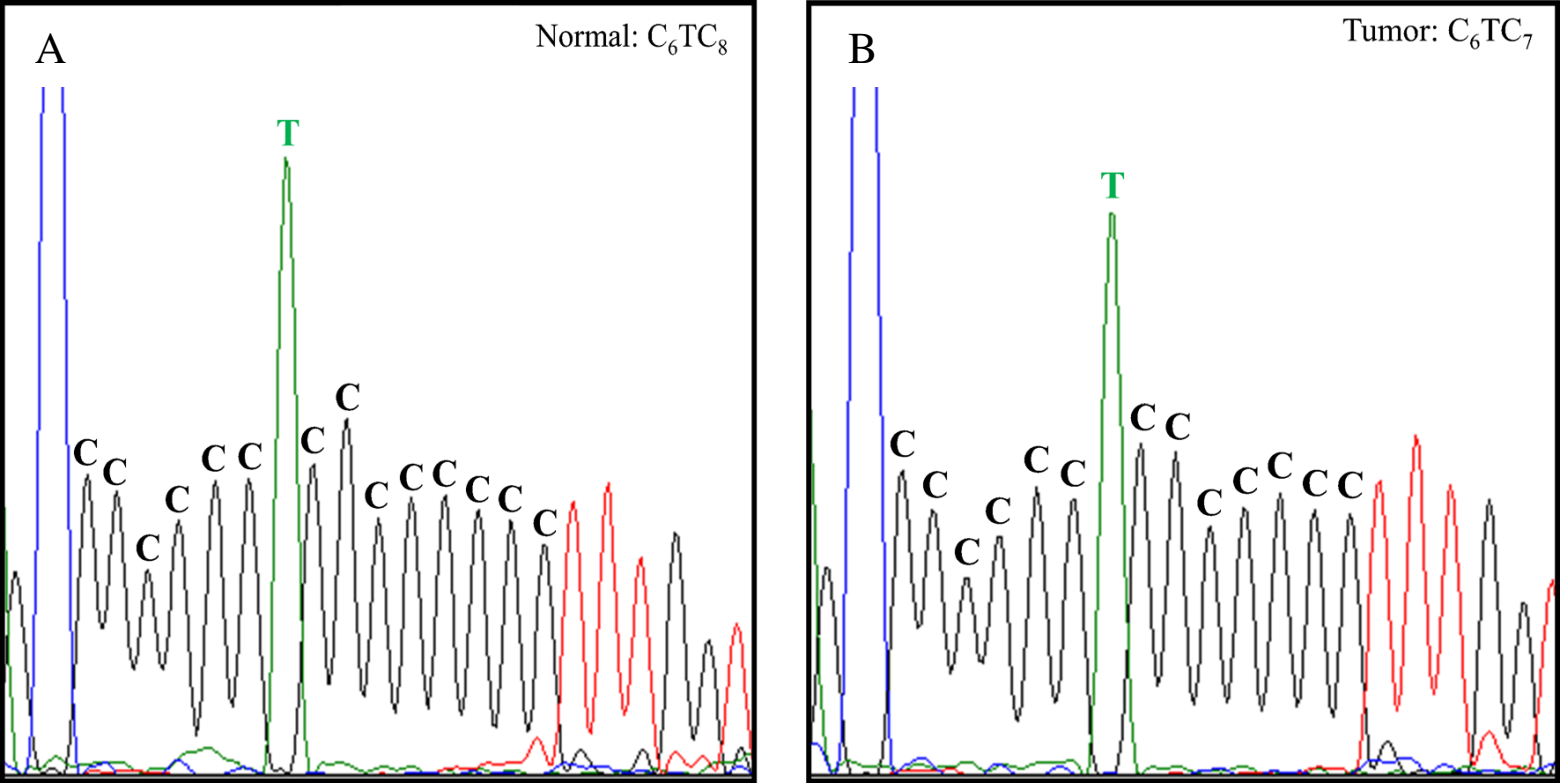
Electropherograms obtained by direct sequencing of the mitochondrial D310 region of matched normal (**a**) and tumor (**b**) tissue. The (C)_n_ status of the major population differ in the samples. **a** C_6_TC_8_ and **b** C_6_TC_7_. This is an example of mtMSI

**Fig. 2:**
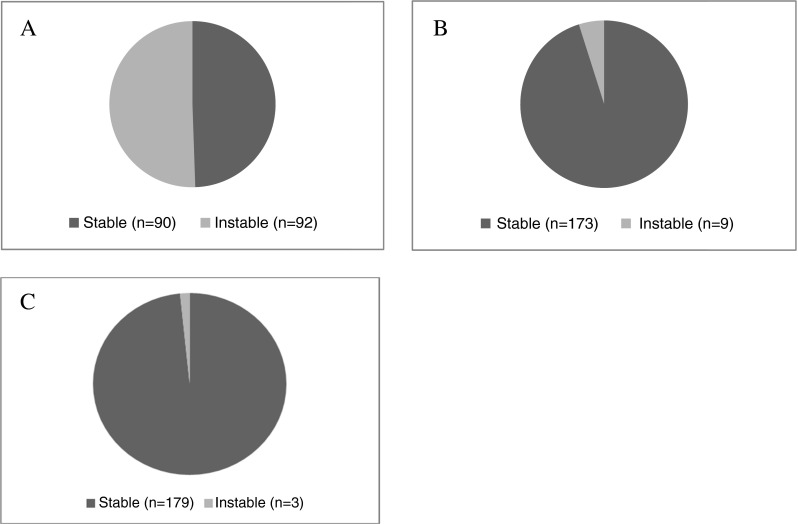
Prevalence of mtMSI at the D310 locus (50.5 %) (**a**), at the D514 locus (4.9 %) (**b**), and at the D18164 locus (1.6 %) (**c**) of all patients

mtMSI has been defined as change in length in short base repetitive sequences of mtDNA between normal and tumor tissue. This assumes that the normal colon tissue of CRC patients has a constant sequence pattern. This was checked for the D310 and D514 region in 10 patients from our hospital database for which we could obtain normal tissue from proximal and distal locations relative to the tumor. We analyzed the mtDNA with PCR and Sanger sequencing. In these 10 patients, we did not observe a difference in the normal tissues, but in five patients, the tumor did show an altered fragment length at the D310 region (Supplementary Table 2). This result confirms that mtMSI is a tumor-specific event and that the comparison of one tumor and one normal tissue block per patient should be sufficient to assess mtMSI. Figure 1 illustrates an example of mtMSI analyzed by direct sequencing. Analyses were done in duplicate. Overall, 54.4 % (99 out of 182) of all patients showed mtMSI at the D-loop region (D310 locus + D514 locus + D16184 locus). The majority of all patients showed mtMSI at the D310 locus (50.5 %), whereas instability at the D514 locus was found in 4.9 % of all patients and instability at the D18164 locus in 1.6 % of all patients (Fig. 2). mtMSI at the D-loop region was most often found at only one of the three D-loop loci (94 out of 99). Five patients showed instability at two loci, with the following combinations: D310 and D514 (*n* = 2), D310 and D16184 (*n* = 2), and D514 and D16184 (*n* = 1). Patients with MSI tumors showed slightly more often mtMSI at the D-loop region compared to patients with MSS tumors; 57.8 versus 51.5 %, *p* = 0.083.

### Prevalence of mtMSI at ND1, ND5, and COX1

Instability at the NADH dehydrogenase subunits ND1 and ND5 was rare. Only 1.1 % of patients (2 out of 182) showed instability at the ND1 locus, and 4.6 % (7 out of 153) of patients showed instability at the ND5 locus. None of the patients showed instability at the cytochrome oxidase subunit COX1.

### Prognosis of mtMSI at the D-loop region

Baseline patient and tumor characteristics of all patients are summarized in Table 1. Survival data for mtMSI at the D-loop region (D310 locus + D514 locus + D16184 locus) are presented in Table 2. There was no correlation of OS with mtMSI at the D-loop region, when analyzing the three D-loop loci separately or together.

**Table 1:** Baseline patient and tumor characteristics of all patients treated in the CAIRO studies, subdivided by patients with MSI and MSS tumors

	Patients with MSI tumors	Patients with MSS tumors	
		Test group	Validation group
	(*n* = 44)	(*n* = 56)	(*n* = 43)
Median age (range)	68 (34–78)	66 (34–79)	68 (39–81)
Sex			
Male	24 (55 %)	37 (66 %)	28 (65 %)
Female	20 (45 %)	19 (34 %)	15 (35 %)
WHO performance status			
PS0	28 (64 %)	56 (100 %)	26 (61 %)
PS1	14 (32 %)	–	16 (37 %)
PS2	2 (4 %)	–	1 (2 %)
Serum LDH			
Normal	32 (73 %)	56 (100 %)	29 (67 %)
Abnormal	12 (27 %)	–	14 (33 %)
Localization of the primary tumor			
Colon	38 (86 %)	48 (86 %)	26 (61 %)
Recto sigmoid	2 (4 %)	8 (14 %)	1 (2 %)
Rectum	3 (7 %)	–	15 (35 %)
Multiple tumor	1 (3 %)	–	1 (2 %)
Metastatic sites involved			
Median	2	2	1
1	16 (36 %)	26 (46 %)	22 (51 %)
>2	26 (60 %)	30 (54 %)	21 (49 %)
unknown	2 (4 %)	–	–
Predominant localization of metastases	–		
Liver	14 (32 %)	40 (71 %)	27 (63 %)
Liver + other	13 (30 %)	–	–
Extra-hepatic	16 (36 %)	15 (27 %)	16 (37 %)
Unknown	1 (2 %)	1 (2 %)	–
Histology of the primary tumor			
Mucinous (>50 % WHO)	16 (36 %)	5 (9 %)	4 (9 %)
Adenocarcinoma with mucinous component	4 (9 %)	6 (11 %)	4 (9 %)
Adenocarcinoma	23 (52 %)	41 (73 %)	34 (80 %)
Unknown	1 (3 %)	4 (7 %)	1 (2 %)

*MSI* microsatellite instability, *MSS* microsatellite stable

**Table 2:** Survival data of the patients with MSI and MSS tumors in relation to the mtMSI status of the D-loop region (D310 locus + D514 locus + D16184 locus)

			D-loop region	
			mtMSS	mtMSI
MSI tumors		Number of patients	17	27
	OS	Months (95 % CI)	16.2 (8.5–19.8)	13.1 (7.3–19.8)
		HR (95 % CI)	0.96 (0.50–1.84)	
Test group (MSS tumors)		Number of patients	30	26
	OS	Months (95 % CI)	19.1 (17.1–28.6)	20.1 (13.6–26.2)
		HR (95 % CI)	1.20 (0.67–2.12)	
Validation group (MSS tumors)		Number of patients	18	25
	OS	Months (95 % CI)	16.3 (12.5–27.3)	18.3 (12.9–21.0)
		HR (95 % CI)	1.23 (0.64–2.35)	

*OS* overall survival, *HR* hazard ratio

### Review of the literature

**Fig. 3:**
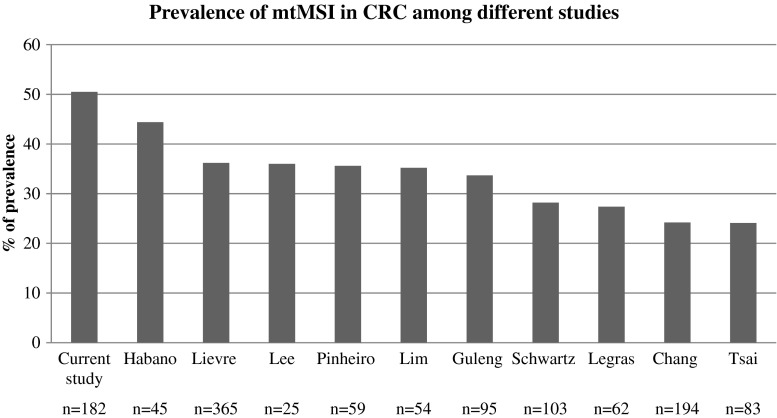
Prevalence of mtMSI at the D310 locus at the D-loop in stage I--IV colorectal cancer patients among different studies

**Fig. 4:**
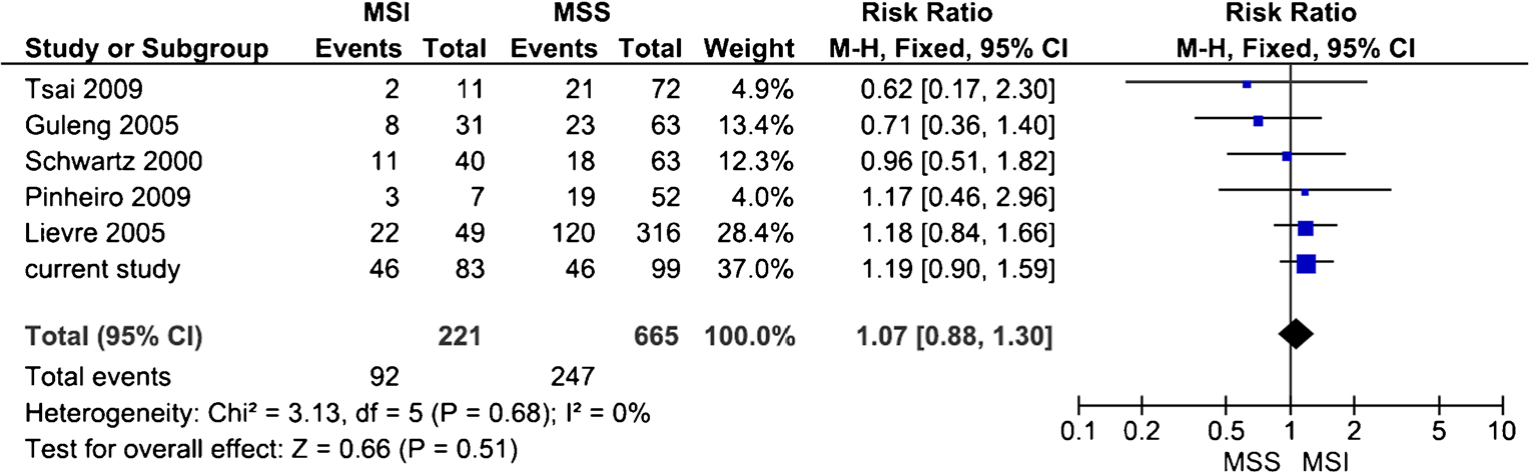
Forest plot of the association of prevalence of mtMSI at the D310 locus in stage I–IV CRC patients with MSI compared to MSS tumors

The literature search identified 10 retrospective, non-randomized, single center studies in stage I–IV CRC (Fig. 3) [6, 11, 12, 24, 26–28, 34, 39, 43]. All studies analyzed the same loci and the same repeat; however, the definition of mtMSI was different among studies. Some studies used the same definition of mtMSI as we used in the current study [11, 12, 28, 39, 43]. While other studies describe insertion and deletions of C nucleotides in the mononucleotide repeat as mutations or alterations [6, 24, 26, 27, 34]. Two studies used band-shifting [12, 39], while all the other studies used direct sequencing to detect mtMSI. All studies evaluated the D310 locus separately. Prevalence of mtMSI at the D310 locus ranged from 24.1 to 44.4 % (Fig. 3). Only one study evaluated the prevalence at other regions (D514 and D16184) of the D-loop, and they found mtMSI at the D16184 locus in 18.6 % of patient and at the D514 locus in 7.4 % of patients [28]. Prevalence of mtMSI at the ND1 and ND5 locus was low and ranged from 1.2 to 6.8 % [12, 34, 43]. In Fig. 4, the different studies comparing the prevalence of mtMSI in stage I–IV CRC patients with MSI and MSS tumors are summarized in a forest plot [11, 27, 34, 39, 43]. mtMSI was not significantly more frequent in tumors with MSI (RR 1.07, 95 % CI 0.88–1.30).

## Discussion

In the current study, we demonstrate a high prevalence of mtMSI in mCRC patients with both MSI and MSS tumors. mtMSI was particularly present at the D310 locus. There was no correlation with prognosis. The D-loop is a triple stranded structure, and its attachment to the nuclear membrane with the subsequent exposure to lipid peroxides formed in the inner mitochondrial membrane makes this region prone for mutations in cancer cells (in particular the D310 region [29]). It is the most variable region in mtDNA and alterations, such as point mutations, insertions, deletions, and mtMSI are common events [3]. This is in line with the high prevalence of instability found at the D310 locus in the current study and previous published studies in stage I–IV CRC (Fig. 2a) [6, 11, 12, 24, 26–28, 34, 39, 43]. In our study, the prevalence of instability at the D-loop (and the D310 locus) is even higher compared to previous published studies [19, 24, 26–33]. The high prevalence might be caused by our selection of patients with metastatic disease, since some studies demonstrated a link between mtMSI with poor prognosis [27] and advanced stage of disease [18, 20]. Alternatively, the variation of prevalence of instability at the D-loop might be caused by differences in the applied definitions and diagnostic panels. There are multiple potential pitfalls in the detection methodology [36]. Direct sequencing has a limited detection level and is hardly quantitative. The heteroplasmy makes it hard to determine whether an alteration results from genuine mutation or simply reflects changes in the proportion of mtDNA subpopulations during mitochondrial segregation. For future research, next generation sequencing and analyses of larger cohorts will be useful. Instability at the ND1 and ND5 genes, subunits of the NADH dehydrogenase complex, is rare in CRC [12, 43] and only occurs in combination with instability at the D310 locus. This suggests that these might be secondary effects, similar to those observed in nuclear MSI [31].

The current study and previous studies on the correlation between nuclear MSI and mtMSI in CRC showed that nuclear MSI and mtMSI are independent events, suggesting that MMR enzymes are not involved in the correction of mtDNA base mispairing [11, 27, 34, 39, 43]. This is consistent with the fact that nuclear MMR proteins do not have any mitochondrial localization signal and cannot be detected in mitochondria by immunohistochemical analysis [13]. Furthermore, it was shown that in mitochondria, YB-1 mediates strong MMR activity, which is unrelated to nuclear MMR proteins [7, 32]. Important questions that still remain unanswered are the following: what are the functional consequences of mtMSI, is mtMSI a cause or a consequence of tumorigenesis, and especially, does mtMSI have prognostic value? The D-loop region is a crucial site for replication and expression of the mitochondrial genome because it contains essential transcription and replication elements [30, 45]. For that reason, one may speculate that sequence variations in this region can alter mtDNA replication and transcription and thus mitochondrial function. Then again, most of the variations are in the polymorphic length range and thus unlikely to lead to functional impairment of the mitochondria [38]. The prognostic value of mtMSI was only analyzed in four other studies in stage I–IV CRC patients, and results were inconsistent [6, 11, 27, 43]. One study found an association between alteration in the D-loop and poor prognosis in stage III CRC patients [27]. However, this was not confirmed in a subsequent study [43]. Also, in our study, there was no correlation with prognosis. Taken together, mtMSI at the D310 locus is frequent in CRC patients, but the prognostic value is still unclear.

## Electronic supplementary material

Below is the link to the electronic supplementary material.

ESM 1 (DOCX 14 kb)

ESM 2 (DOCX 14 kb)
