# Supplementary material for: Mitochondrial microsatellite instability in patients with metastatic colorectal cancer
Source: Virchows Arch. 2015 Feb 20;466(5):495–502. doi: 10.1007/s00428-015-1733-8 (PMC4422840; doi:10.1007/s00428-015-1733-8)
Supplement: Supplementary file 1 — (DOCX 14 kb) [file 428_2015_1733_MOESM1_ESM.docx]

Supplementary Table 1. Primer sequences of used mtMSI primers

| Name primer | Primer | Size |
| --- | --- | --- |
| Repeat | sequence | amplicon |
| D-loop 310 | **ACA GCC GCT TTC CAC ACA G**AC ATC ATA ACA AAA AAT TTC CAC CAA ACC CCC CCC TCC CCC CGC TTC | 133 |
| C_8_TC_6_ | TGG CCA CAG CAC TTA AAC ACA TCT CTG CCA AAC CCC AAA AAC AAA GAA **CCC TAA CAC CAG CCT AAC CA** |  |
| D-loop 514 | **CCC ATA CTA CTA ATC TCA TCA A**TA CAA CCC CCG CCC ATC CTA CCC AGC ACA CAC ACA CCG CTG CTA | 89 |
| CA_5_ | ACC C**CA TAC CCC GAA CCA ACC AAA** |  |
| D-loop 16184 | **CTT GAC CAC CTG TAG TAC ATA** AAA ACC CAA CCC ACA TCA AAC CCC CCC CCC CCA TGC TTA CAA | 110 |
| C_12_ | GCA AGT ACA GCA ATC AAC CTT CAA CTA **TCA CAC ATC AAC TGC AAC TCC** |  |
| ND1 | **CCG ACC TTA GCT CTC ACC AT**C GCT CTT CTA CTA TGA ACC CCC CTC CCC ATA CCC AAC | 88 |
| C_6_ | CCC CTG GTC AAC **CTC AAC CTA GGC CTC CTA TT** |  |
| ND5 | **CAC CCT AAC CCT GAC TTC C**CT AAT TCC CCC CAT CCT TAC CAC CCT CGT TAA CCC TAA CAA AAA AAA | 105 |
| C_6_A_8_ | CTC ATA CCC CCA TTA TGT AA**A ATC CAT TGT CGC ATC CAC C** |  |
| COX1 | **CCT ACC AGG CTT CGG AAT AA**T CTC CCA TAT TGT AAC TTA CTA CTC CGG AAA AAA AGA | 89 |
| A_7_ | ACC ATT TGG ATA **CAT AGG TAT GGT CTG AGC TAT** |  |
|  | |  |
| Bold: forward and reverse primer | |  |
| Underlined: repeat | |  |
