# Supplementary material for: Mitochondrial microsatellite instability in patients with metastatic colorectal cancer
Source: Virchows Arch. 2015 Feb 20;466(5):495–502. doi: 10.1007/s00428-015-1733-8 (PMC4422840; doi:10.1007/s00428-015-1733-8)
Supplement: Supplementary file 2 — (DOCX 14 kb) [file 428_2015_1733_MOESM2_ESM.docx]

Supplementary Table 2. mtMSI analysis of ten patients for which we have two normal samples

|  | **D310 locus** | | | | **D514 locus** | | | |
| --- | --- | --- | --- | --- | --- | --- | --- | --- |
| Case | Tumor | Normal distal | Normal proximal | mtMSI status | Tumor | Normal distal | Normal proximal | mtMSI status |
| 1 | C7TC6 | C7TC6 | C7TC6 | MSS | C4 | C4 | C4 | MSS |
| 2 | C7TC6 | C7TC6 | C7TC6 | MSS | C5 | C5 | C5 | MSS |
| 3 | C7TC6 | C7TC6 | C7TC6 | MSS | C5 | C5 | C5 | MSS |
| 4 | C7TC6 | C7TC6 | C7TC6 | MSS | C5 | C5 | C5 | MSS |
| 5 | C7TC6 | C7TC6 | C7TC6 | MSS | C5 | C5 | C5 | MSS |
| 6 | C5TC6 + C10TC6 | C7TC6 | C7TC6 | MSI | C5 | C5 | C5 | MSS |
| 7 | C7TC6 | C8TC6 | C8TC6 | MSI | C5 | C5 | C5 | MSS |
| 8 | C7TC6 + C9TC6 | C7TC6 | C7TC6 | MSI | C4 | C4 | C4 | MSS |
| 9 | C8TC6 | C7TC6 + C8TC6 | C7TC6 + C8TC6 | MSI | C5 | C5 | C5 | MSS |
| 10 | C8TC6 + C9TC6 | C7TC6 + C8TC6 | C7TC6 + C8TC6 | MSI | C5 | C5 | C5 | MSS |
